# Col V siRNA Engineered Tenocytes for Tendon Tissue Engineering

**DOI:** 10.1371/journal.pone.0021154

**Published:** 2011-06-21

**Authors:** Ping Lu, Guo Rong Zhang, Xing Hui Song, Xiao Hui Zou, Lin Lin Wang, Hong Wei Ouyang

**Affiliations:** 1 Center for Stem Cell and Tissue Engineering, School of Medicine, Zhejiang University, Hangzhou, China; 2 Institute of Cell Biology, School of Medicine, Zhejiang University, Hangzhou, China; 3 Women Hospital, School of Medicine, Zhejiang University, Hangzhou, China; The University of Hong Kong, Hong Kong

## Abstract

The presence of uniformly small collagen fibrils in tendon repair is believed to play a major role in suboptimal tendon healing. Collagen V is significantly elevated in healing tendons and plays an important role in fibrillogenesis. The objective of this study was to investigate the effect of a particular chain of collagen V on the fibrillogenesis of Sprague-Dawley rat tenocytes, as well as the efficacy of Col V siRNA engineered tenocytes for tendon tissue engineering. RNA interference gene therapy and a scaffold free tissue engineered tendon model were employed. The results showed that scaffold free tissue engineered tendon had tissue-specific tendon structure. Down regulation of collagen V α1 or α2 chains by siRNAs (Col5α1 siRNA, Col5α2 siRNA) had different effects on collagen I and decorin gene expressions. Col5α1 siRNA treated tenocytes had smaller collagen fibrils with abnormal morphology; while those Col5α2 siRNA treated tenocytes had the same morphology as normal tenocytes. Furthermore, it was found that tendons formed by coculture of Col5α1 siRNA treated tenocytes with normal tenocytes at a proper ratio had larger collagen fibrils and relative normal contour. Conclusively, it was demonstrated that Col V siRNA engineered tenocytes improved tendon tissue regeneration. And an optimal level of collagen V is vital in regulating collagen fibrillogenesis. This may provide a basis for future development of novel cellular- and molecular biology-based therapeutics for tendon diseases.

## Introduction

Tendons and ligaments are frequently targets of injury from trauma in sports and aging [1], [2]. It is well known that tendons and ligaments do not heal through a regenerative process; Instead, their healing occurs via the formation of a fibrotic scar [3]–[5]. In the healing tendon, a uniform distribution of small diameter collagen fibrils has been found with poorer mechanical properties than native tissue and shows no improvement of mechanical properties with time[6]. Previous studies have shown that the diameter of collagen fibrils in soft tissues has a positive correlation with collagen mechanical strength [7], [8]. So, the generation of larger diameter fibrils is believed to promote biomechanical properties of healing tendons.

Fibril-forming collagens in most connective tissues include collagen types I, III, and V. Collagen V is a quantitatively minor component of the tissues such as dermis, tendon/ligament and bone. Unlike cornea, where collagen V represents 20 to 25% of the total collagen and such high percentage contributes to their transparence [9], in most above tissues the relative concentration of collagen V is significantly lower, only 1 to 3%. However, collagen V has a key role in the regulation of initial fibril assembly. Type V and Type I collagen are co-assembled into heterotypic fibrils. The entire triple-helical domain of the type V collagen molecules is buried within the fibril and type I collagen molecules are present along the fibril surface [10], [11], and the retained NH2-terminal domains of the type V collagen are exposed at the surface, extending outward through the gap zones. At the early stage of assembly, immature fibril segments is regulated by the NH2-terminal domain of type V collagen [11]. These NH2-terminal domains alter accretion of collagen molecules onto fibrils and therefore lateral growth. A critical density of type V collagen would favor the initiation of new fibrils rather than the continued growth of existing fibrils. Type V collagen molecules may contain α1 (V), α2(V) and α3(V) chains [12], [13]. There are several collagen V isoforms that differ in chain composition, such as α1(V)_2_α2(V) heterotrimer, α1(V)α2(V)α3(V) heterotrimer, α1(V)_3_ homotrimer [14]–[16]. However, collagen fibrils in tendon and ligament are heterotypic type I/V fibrils and α1(V)_2_α2(V) is the favored and functional heterotrimer [16], [17]. Ultrastructural immunolocalization demonstrated that the homotrimer α1(V)_3_ was localized at the surface of wide collagen I fibrils as thin filamentous structures and did not regulate fibril assembly, whereas the heterotrimer α1(V)_2_α2(V) was buried in the fibril interior and regulated collagen fibrillogenesis [18]. Studies have indicated that the occurrence of a uniform distribution of small diameter collagen fibrils [6], along with a persistently elevated level of collagen V, for up to 52 weeks after injury [19]. Several investigations have shown that type V collagen plays a role in regulating the diameter of the type I collagen fibril during fibrillogenesis [10], [11], [20], [21]. The collagen fibril diameter was inversely proportional to type V/type I collagen ratios [23], i.e. the higher concentration of type V, the smaller the fibril diameter [19]. Although the involvement of collagen V in tendon matrix organization is well established, the functional significance of the particular chains of collagen V in tendon tissue engineering is remained unknown.

At present, the models for investigating tendon biology include cell and animal models [21], [22]. Cell models are simple, however, are not convenient for the subsequent investigation of collagen fibril synthesis [22], [23]. Animal models are powerful for detecting collagen fibril synthesis, but contain more influencing factors than *in vitro* study and ethical difficulties also present. Previous studies by our group successfully utilized mesenchymal stem cell sheets assembled with frozen tendon graft for tendon tissue engineering [24]. In this study, a scaffold-free tissue engineered tendon was developed with tenocyte sheets. This provides a simple and controllable 3-D tissue engineering model, which mimics tissue *in vivo* and makes the tendon biological study *in vitro* feasible.

The objective of this study was to investigate the effect of particular chain of collagen V on the fibrillogenesis of tenocytes, as well as the efficacy of Col V siRNA engineered tenocytes for tendon tissue engineering. RNA interference technology is an efficient tool for silencing endogenous or exogenous genes by complementary binding to the target mRNA [25]. Since there are two collagen V chains, therefore, we use siRNA oligonucleotides to decrease the two chains of type V collagen synthesis separately in tissue engineered tendon model as a logical approach to understanding the role of type V collagen during the tendon healing process.

## Results

### Gene expression of injured and normal tendon tissue

**Figure 1:**
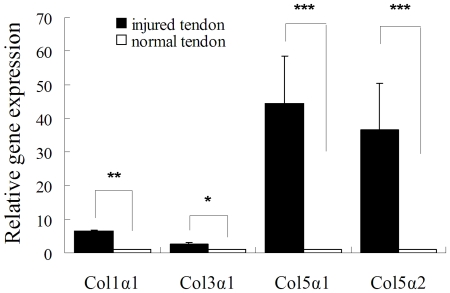
Gene expression profiles of injured tendon at 1 week after injury and normal tendon tissue (*P<0.05, **P<0.01, ***P<0.001 compared with normal tendon group, n = 3).

To verify the expression of different chains of collagen V in the injured tendon, real time PCR was used to detect their mRNA expression 1 week after surgery and compare with that of normal tendon tissue. The results showed that injured tendon had significant higher expression levels of Col1α1, Col3α1, Col5α1 and Col5α2 than those in normal tendon tissue (Fig. 1, P <0.05 or P<0.01), whereas those of Col5α1 and Col5α2 were much higher, so we choose these two for further study.

### Morphology and biochemical properties of the *in vitro* tissue engineered tendon

**Figure 2:**
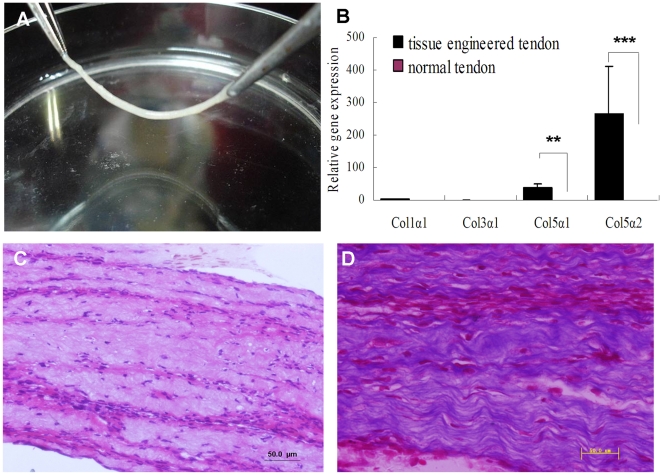
Morphology and biochemical properties of tissue engineered tendon. (A) Construction of *in vitro* tissue engineered tendon. (B) Gene expression of tissue engineered tendon in contrast to normal Achilles tendon tissue. (C, D) Histological examination and Masson trichrome staining of tissue engineered tendon (**P<0.01, ***P<0.001 compared with normal tendon group, n = 3).

Rat Achilles tenocytes proliferated rapidly and formed coherent cellular sheets within 2 weeks after attaining confluence in the presence of ascorbic acid. After the sheets formed, they could be detached from the culture substratum, so a tissue engineered tendon with living cells and collagen matrix which had a relatively compact structure was obtained (Fig.2A). Hematoxylin-eosin and Masson trichrome staining of tissue engineered tendon revealed a tissue-specific tendon structure: organized bundles of highly crimped fibrils and cells oriented in parallel (Fig. 2C, D). And the real time PCR results showed that the cultured cell sheet had high level of collagen V expression (Fig. 2B).

### Transfection of siRNA against collagen V efficiently suppressed collagen V expression in rat Achilles tenocytes

**Figure 3:**
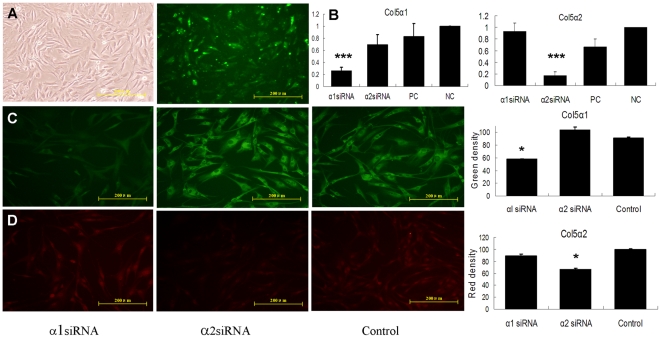
Transfection of siRNA against collagen V strongly suppressed collagen V expression in rat Achilles tenocytes. (A) Transfection efficiency of FAM-labeled scrambled siRNA delivery. (B) Gene expression at 48 h after transfection. In each case, negative control values were set to 1.0 and the rest of the values normalized to it. The housekeeping gene β-actin was amplified as an internal control. The results are expressed as mean ±SD. PC, positive control: GAPDH siRNA; NC, negative control: scrambled siRNA (*P<0.05, ***P<0.001 compared with negative control group, n = 3). (C, D) Representative immunostaining for Col5α1 and Col5α2 at 48 h after transfection. Control: Cells treated with transfection agent only. The value represented the mean color density of each cell.

We transfected two silencer pre-designed siRNA sequences against different chains of collagen V into rat achilles tenocytes by Lipofectamine 2000. As demonstrated by fluorescent microscopy, uptake of the siRNAs by rat Achilles tendon was highly efficient. The green fluorescent signal was observed as early as 1 h after transfection and reached a maximum at 6 h and could still be seen 48 h after the application of oligonucleotides (Fig. 3A).

Forty-eight hours after transfection, real time PCR analysis revealed siRNA196227 (Col5α1 siRNA) and siRNA s136862 (Col5α2 siRNA) led to remarkable suppression of Col5α1 and Col5α2 expression respectively compared to the negative control group (NC: scrambled siRNA). There was at least ∼70% knockdown of collagen V mRNA (Fig. 3B, P<0.05). The positive control (PC: GAPDH siRNA) also had evident suppression of GAPDH (data not shown). The positive control was used to verify the methods of siRNA transfection. Taken together, we chose siRNA 196227 and siRNA s136862 in the subsequent experiments.

As shown in the collagen V immunofluorescence staining results (fig. 3C, D), tendon cells had decreased fluorescence in response to particular siRNA interference, which indicates down-regulation of Col5α1 and Col5α2 at the protein level. The control group (cells treated with transfection agent only) showed high level of fluorescence indicating constitutively high levels of type V procollagen α1 and α2 expression, while Col5α1 siRNA decreased the expression level of Col5α1 by ∼36.6%, and Col5α2 siRNA down-regulated the level of Col5α2 by ∼33.7%. Such findings were consistent with the mRNA expression results. These data collectively indicate that collagen V was efficiently down-regulated at the mRNA and protein levels by siRNA interference methods.

### Effect of different chain of collagen V on cell proliferation and apoptosis

**Figure 4:**
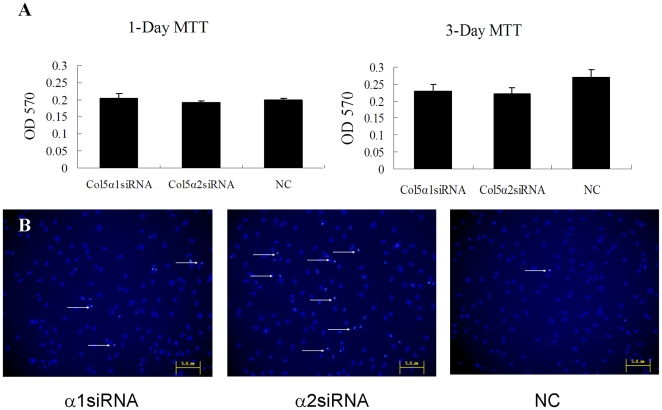
Effect of down regulation of different chain of collagen V on cell proliferation, apoptosis and collagen synthesis. (A) Cell proliferation treated with siRNA at 1 day or 3 days. (B) Apoptosis with DAPI staining at 3 days after transfection.

The MTT assay for tendon cell viability revealed that both of the siRNA treatments caused little difference between the groups at day 1 after transfection. The reduction in OD_570_ was statistically significant for cells treated with Col5α1 siRNA and Col5α2 siRNA (Fig. 4A, P<0.05) at day 3. The mean ± SD OD_570_ was 0.229±0.0208 and 0.221±0.018 for cultures treated with Col5α1 siRNA and Col5α2 siRNA, respectively. While the OD_570_value in control cells was 0.271±0.0216. These results clearly indicated that down regulation of both chains of collagen V had a modest negative influence on the viability of tendon cells.

In DAPI staining, the labeled DNA of viable cells was scattered throughout the nuclei, and bright condensation of chromatin revealed apoptotic cells. A few more apoptotic cells were found in the Col5 siRNA group (Fig. 4B). These results indicate that the lower growth rate of cells treated with COL5 siRNA may have been caused by the increased apoptosis when the collagen V was down-regulated (26).

### Different chains of collagen V have different effects on fibrillogenesis and collagen fibril diameter

**Figure 5:**
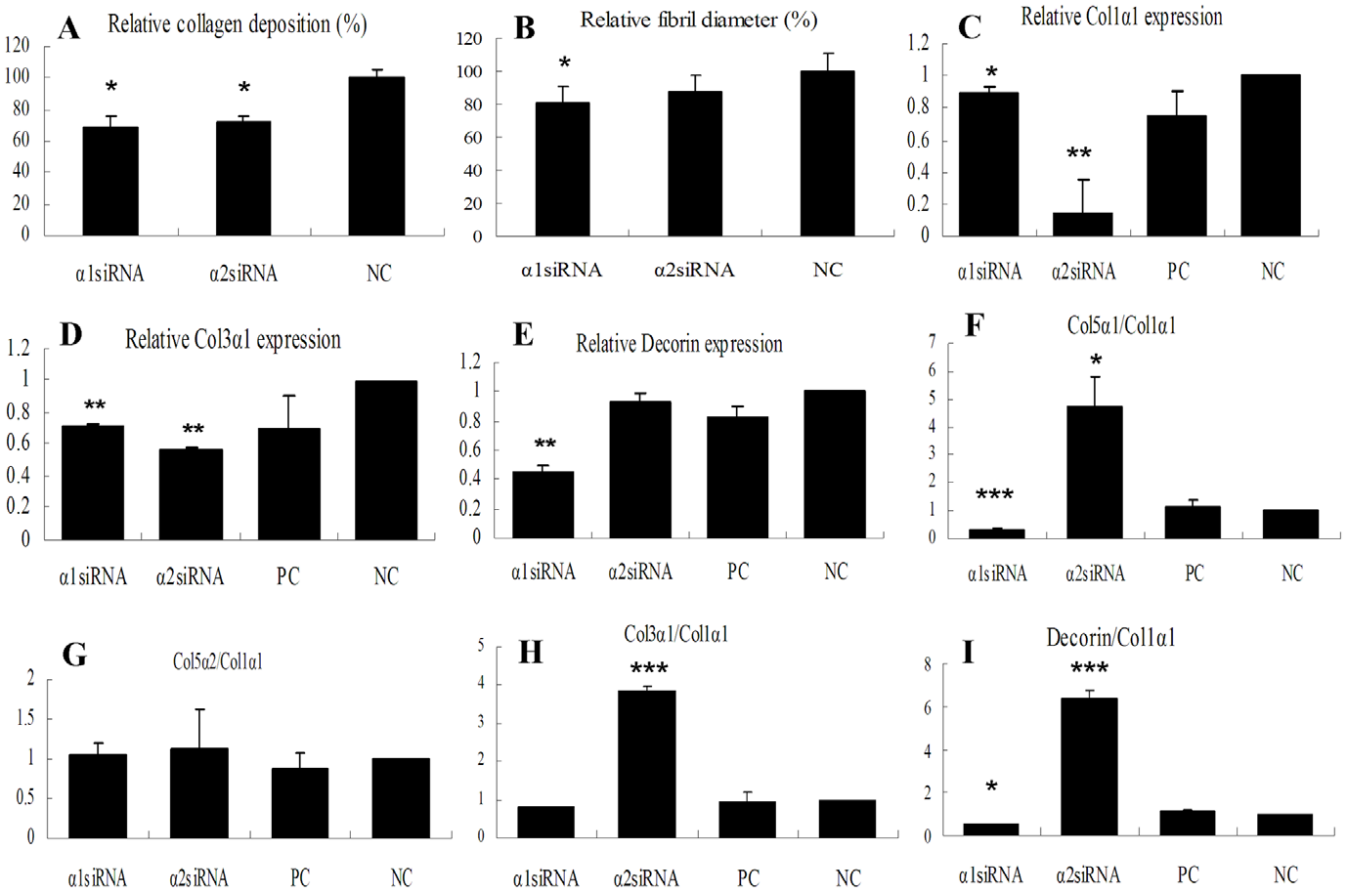
Effects of siRNA treatment on the extracellular matrix (ECM) deposition and collagen fibril diameter. The influence of siRNA treatment on relative collagen deposition (A) and relative mean diameter (B) (* P<0.05 compared with negative control group, n = 3). The influence of siRNA silencing on the expression of some ECM genes and their proportions to collagen type I in tissue engineered tendon (C: Col1 α1; D: Col3 α1; E: Decorin; F: Col5 α1/ Col1 α1; G: Col5 α2/Col1 α1; H: Col3 α1/Col1 α1; I: Decorin/Col1 α1), (*P<0.05, **P<0.01, ***P<0.001 compared with normal tendon group, n = 3).

The amount of collagen deposited in the tissue engineered tendon was shown in Fig. 5A. The mean ± SD of collagen deposition was (35.1±7.3) % and (38.9±6.8) % of the value in control cells for cultures treated with Col5α1 siRNA and Col5α2 siRNA, respectively (P<0.05). There was less of a difference between the two experimental groups. The results indicated that down-regulated collagen V impaired collagen expression in Achilles tenocytes.

Transmission electron microscopy (TEM) results showed that the *in vitro* tissue engineered tendon had organized collagen fibrils. However, tissue engineered tendon treated with Col5α1 siRNA had abnormal fibril ultrastructure, while the fibrils from tendon treated with Col5α2 siRNA was similar to that of the control (data not shown). Comparison of mean fibril diameters in different treatment are shown in Fig. 5B. The relative mean diameters of α1 siRNA and α2 siRNA treatments were about (80.6±10.06)% and (87.3±10.11)% of the control.

### Different chains of collagen V have different effects on matrix gene expression

To clarify the molecular events after blocking collagen V expression, we also assessed the expression of some important collagen and decorin genes by real time PCR and compared their proportions to collagen type I. The expression of Col1α1 was down regulated when either siRNA was added, especially reduced by Col5α2 siRNA to ∼16.5% (Fig. 5C, P<0.01). Quantitive PCR analysis also showed both collagen type III and decorin mRNA levels were decreased after dealing with Col5α1 siRNA or Col5α2 siRNA (Fig. 5D and F). These finding indicate that the block of Col V may affected the synthesis of these extracellular matrix.

In the Col5α1 siRNA treated group, the ratios of Col5α1/Col1α1, Col3α1/ Col1α1 and decorin/Col1α1 were sharply decreased by 70.5%, 20.6% and 48.7%, respectively. In the Col5α2 siRNA treated group, the ratio of Col5α2/Col1α1 was decreased by 68.5%, whereas the ratios of Col5α1/Col1α1 and decorin/Col1α1 were increased by 30.7% and 75.9%, respectively (Fig.5 G–I). These results indicated that Col5α1 siRNA and Col5α2 siRNA have different function in regulating matrix gene expression at the mRNA level, and may indirectly lead to different effects on collagen synthesis and tendon repair.

### Transmission electron microscopy analysis of tissue engineered tendons formed by coculture of Col5α1 siRNA treated tenocytes and normal tenocytes in different ratios

**Figure 6:**
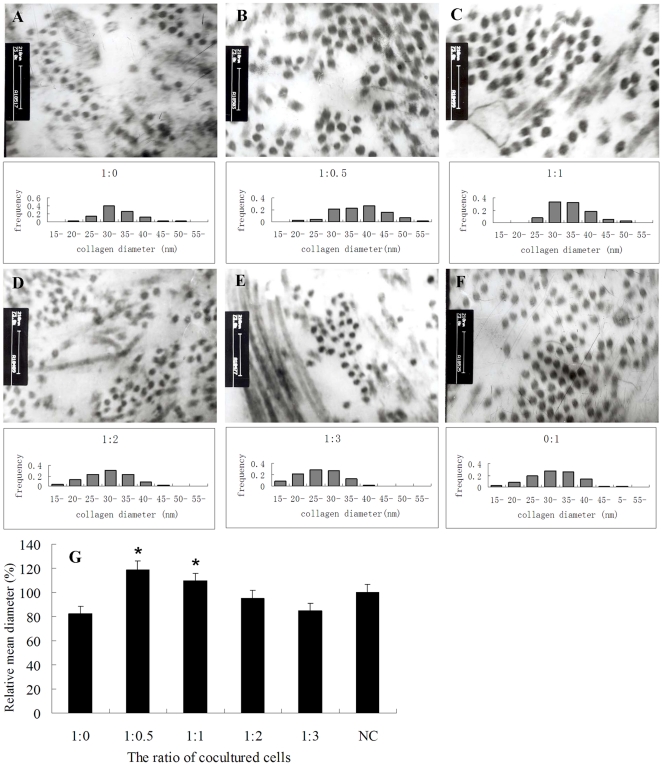
Transmission electron microscopic analysis and histogram of collagen fiber diameters of tissue engineered tendons formed by coculture of Col5α1 siRNA treated tenocytes and normal tenocytes at different ratios [A, 1∶0; B, 1∶0.5; C, 1∶1; D, 1∶2; E, 1∶3; F, 0∶1 (NC), magnification 73000×]. (G) The relative mean diameters of tissue engineered tendons formed by cocultured cells at different ratios (* P<0.05 compared with negative control group, n = 3).

Since tissue engineered tendons treated with Col5α1siRNA had abnormal collagen fibrils, a possible explanation is that the corresponding chain of collagen type V is over-suppressed. So we cocultured Col5α1 siRNA treated tenocytes and normal tenocytes with ratios of 1∶0, 1∶0.5, 1∶1, 1∶ 2, 1∶3 and 0∶1 (NC group). As shown in Fig.6 (A–F) are their transmission electron microscopic analysis and histogram of collagen fiber diameters of tissue engineered tendons. Compared with collagen fibrils of the control group, collagen fibrils of cocultured groups with a ratio of 1∶0.5 or 1∶1 had similar morphology and relative larger diameters with the mean diameter (118.8±7.52)% and (109.8±5.78)% (Fig.6G) of the control, respectively. While collagen fibrils of cocultured groups with a ratio of 1∶2 or 1∶3 were (94±6.56)% and (84.9±5.83)% (Fig.6G) of the control group. Those results indicate that regulating the quantity of collagen type V α1 to a proper level is beneficial for the generation of larger diameters.

## Discussion

The present study for the first time demonstrated the use of a scaffold-free tissue engineered tendon model for investigating the biological function of collagen V in tendon fibrillogenesis. We found that the type V procollagen α1 and α2 chains had different effects on regulating tendon matrix gene expression to some extent and an optimal level of collagen type Vα1 is vital in regulating collagen fibrillogenesis. Col V siRNA engineered tenocytes improved tendon tissue engineering. These findings present a good example of in vitro tissue engineering model for tendon biology investigation and may provide basis for future development of cell or gene therapy for tendon repair.

The basic function of tendon is to transmit force from muscle to bone, which makes limb and joint movement possible. Therefore tendons must be capable of resisting high tensile forces with limited elongation. Parry and his colleagues reported that the mechanical properties of tendons are related to the fibril diameter distribution, large fibrils could withstand higher tensile forces [8]. Moreover, investigations have also showed that a greater fibril diameter generates greater tensile strength and the relative smaller fibril diameter provides greater elastic properties, which is of consistence to the region-specific function of patellar tendon. So for the tendon to function properly it seems that there is need for both small and large diameter fibrils [27], [28]. However, during the process of repair, collagen fiber diameter is notably smaller compared to the normal health one and the larger fiber in the health tendon occupy the most volume, so now for tendon repair, regeneration of larger diameter is one of the major challenge.

### Tissue engineering tendon model for biological studies of tendon

Previous studies of tendon biology used either *in vitro* cell cultures or animal models [21], [22], but both of them have several inherent disadvantages. In our study, a relative well-organized collagen fibrils resembling tendon ultrastructure is formed. Meanwhile, the tissue engineered tendon is scaffold-free, so there are fewer exogenous factors. It can be easily and efficiently used to evaluate the role of gene intervention (e.g. RNAi and antisense techniques) in tendon research. In addition, our tissue engineered tendon is an excellent setup to simulate injured tendon. It has a uniform distribution of small diameter collagen fibrils along with a high level of type V collagen expression, which is characteristic of injured tendon [6], [19]. Although the tissue engineered tendon lacks a fair bit in the mechanical perspective and has less efficient fibrillogenesis, it still illustrates a novel model and strategy for future tendon biology research and studies.

### Effects of different chains of collagen V on tendon fibrillogenesis

This study assessed the function of two chains of collagen V. The amount of collagen deposited in the tissue engineered tendon indicated that down-regulated collagen V impaired collagen formation in Achilles tenocytes. The down-regulation of Col I expression and consequently possible affected Col I turnover may be one of the reasons that contributed to the collagen deposition decrease. Moreover, it seems that the influence of Col5α1 on fibril formation was more apparent than that of Col5α2. Tissue engineered tendon treated with Col5α1 siRNA had abnormal collagen fibril morphology while tendon treated with Col5α2 siRNA had collagen fibrils similar to controls. Published data indicate that cells deficient in Col5α1 would be null for type V collagen since all three isoforms contain this chain, while cell deficient in Col5α2 would be expected to assemble the α1(V)_3_ homotrimer. However, unlike the heterotrimer α1(V)_2_α2(V), the homotrimer α1(V)_3_ was shown to not assemble into heterotypic type I/V fibrils and hence did not participate in collagen fibrils formation [18]. So, the difference of collagen fibrillogenesis between Col5α1and Col5α2 siRNA treatments may be caused by the over-depression of Col5α1. Real time PCR results indicated that Col5α1 siRNA and Col5α2 siRNA had different effects on the expression of Col1α1 and decorin at the mRNA level. Col5α1 siRNA decreased the ratios of Col5α1, Col3α1 and decorin to Col1α1, whereas Col5α2 siRNA increased the ratios of Col5α1, Col3α1 and decorin to Col1α1. Collagen type I is the major quantitative component in collagen fibrils, whereas Col5α1 and Col3α1 are two minor quantitative contents. The ratios between these two genes and Col1α1 are reversely related to the fibril diameter [29]–[33]. Thus, it is a paradox since all the ratio values were decreased while the fibrils diameter is also smaller in the Col5α1 siRNA group. Over silencing of Col5α1 may be the answer for such contradiction. The sharp reduction of collagen V and decorin by Col5α1 siRNA may could not assemble enough α1(V)_2_α2(V) functional isoform and proteoglycan to form V/I heterotypic fibrils and collagen-proteoglycan heteropolymers to fulfill the minimal requirement of collagen I fibril formation [23]. In the Col5α2 siRNA-treated group, those ratios are consisted with the diameters. Decorin belongs to small leucine-rich proteoglycans/proteins (SLRPs) and the formation of collagen-proteoglycan heteropolymers is important in regulation of fibrillogenesis [34]–[37]. Whether the ratios of decorin to collagen type I may also be inversely proportional to collagen fibril diameter is needed to be determined.

Collagen formation processes are driven by the loss of solvent molecules in cell-surface crypts [38] and result in assemblies with a circular cross-section. Type V and type I collagen co-assemble into heterotypic fibrils and regulate the initial fibril assembly. This assembly is completely processed outside the cell [39]. Over-depression of collagen V in tissue engineered tendon using Col5α1 siRNA maybe caused small and abnormal collagen fibrils, so we cocultured Col5α1 siRNA treated tenocytes and normal tenocytes with different ratios. The coculture experimental results revealed that collagen fibrils of cocultured groups with a ratio of 1∶0.5 or 1∶1 had similar morphology and larger diameters, while collagen fibrils of cocultured groups with a ratio of 1∶0, 1∶2 or 1∶3 were smaller. These results indicated that there was an optimal level of collagen type V and the level was vital in regulating collagen fibrillogenesis. The undetermined precise level of collagen type V is the limitation of the present study and warrants further investigation to obtain. Although the mechanisms involved need further investigation, and the effects of both chains blocked should be studied, these findings provided useful information for the development of molecular biology- and cell-based therapeutics for improving injured tendon repair in future.

### Effect of different chains of collagen V on cell proliferation and apoptosis

Our results in siRNA treated tendon cells suggest collagen V involvement in biological processes besides fibrillogenesis. A few more Col5α2 siRNA-treated cells were DAPI positive and exhibited morphological signs of apoptosis, as judged by fluorescence microscopy, while Col5α1 siRNA had a modest negative influence on cell growth and apoptosis. The apoptosis of Col5α2 siRNA-treated cells may be a consequence of the loss of important cell-matrix interactions. This finding is in line with the established role of the extracellular matrix in sustaining cell survival. Changes in matrix composition may affect the adhesion signals of cells [26] and the storage and activation of growth factors [40]. Previous studies using mice harboring a targeted deletion of the Col5α2 gene have shown increased apoptosis of stromal fibroblasts in skin [26].

### RNAi for tendon matrix gene regulation

This study showed that siRNA interference successfully inhibited procollagen α1 and α2 (V) synthesis in rat Achilles tendon fibroblasts. RNAi is the process of sequence specific, post-transcriptional gene silencing directed by short interfering 21–23 nt double-stranded RNA (siRNA) [41]. siRNAs act as guides to activate the RNA-induced silencing complex, which cleaves homologous mRNA molecules [25]. Many studies have demonstrated that the introduction of siRNAs into mammalian and human cells causes specific and effective suppression of the corresponding mRNA molecules [25], [41]–[43]. Several lines of evidence that siRNAs inhibit the *in vivo* expression of endogenous genes provides further support for the notion that specific siRNAs may be a new alternative to gene-specific therapeutics of human diseases [43]. For the therapeutic application of siRNA technology, it is critical to employ an efficient gene delivery system for transduction of siRNA into target cells.

In conclusion, analysis of the different chains of collagen type V shed light on the role of this minor collagen type in fibrillogenesis and tendon regeneration. Our experiments document the critical contribution of the α1 (V) and α2 (V) chains to fibrillogenesis, fibril organization, and cell viability. In addition, this work successfully used scaffold-free tendon tissue engineered model to mimic tissue *in vivo* for investigating the biological process of collagen fibrillogenesis. This provides a simple and controllable model for future tendon biology studies. Meanwhile, the optimal level of collagen V in injured tendon repair is vital and its details need further investigation.

## Materials and Methods

### Cell culture

Primary tenocytes were isolated from the Achilles tendon of Sprague-Dawley rats. The Protocols were approved by the animal care and use committee of Zhejiang University. The ethical grant number is zju2007102001. In brief, Achilles tendons were harvested and adjacent tissues were stripped. After soaking in phosphate-buffered saline (PBS) containing penicillin (100 units/ml) and streptomycin (100 mg/ml) (Gibco, Carlsbad, CA, USA) for 10 min, the tendon was cut into small fragments followed by enzyme digestion with a 0.2% (W/V) collagenase mixture (Gibco, Carlsbad, CA, USA) at 37°C in a humidified atmosphere of 5% CO_2_ and 95% air. After 4 h, an equal volume of Dulbecco's modified Eagle medium (DMEM) containing 10% (V/V) fetal bovine serum (FBS; Gibco, Carlsbad, CA, USA) was added and the mixture digested overnight. Next day, the tissue residue was removed and the resulting cell suspension was centrifuged at 1,200 rpm. The harvested cells were maintained in DMEM containing 10% (V/V) FBS with medium changed every 2–3 days. When cultured primary cells reached 80% confluence, they were detached by treatment with 0.25% (W/V) trypsin and 0.1% ethylenediaminetetraacetic acid (Gibco, Carlsbad, CA, USA) and subcultured at a density of 1 × 10^4^/cm^2^. Cultured cells before passage 5 were used for experiments.

### siRNA synthesis and transfection

Two target sequences (Table 1) for mRNAs of the α1 and α2 chains in rat collagen type V were chosen according to the online Ambion siRNA design tool. The specificity of all sequences was confirmed by BLAST search. All siRNAs were synthesized by Ambion Co. (Austin, TX, USA). The positive control (PC, targeting the housekeeping gene glyceraldehyde 3-phosphate dehydrogenase, GAPDH siRNA) and FAM-labeled negative control (NC, scrambled siRNA) siRNAs were from Ambion. Transfection of siRNA was performed with Lipofectamine 2000 (Invitrogen, Carlsbad, CA, USA) according to the manufacturer's protocol.

**Table 1:** siRNA sequences of Col5α1 and Col5α2.

Si RNA ID	Target Chain	Oligonucleotide Sequence (5′-3′)
196226	α1	GCAGCUGUACCCUGAGUCUtt
196227	α1	CGAUGAGGAAAUGUCUUAUtt
s136861	α2	GUCUCUCAGUAGUCAAAUUtt
s136862	α2	CACUGUUGGUUAUAUGGAUtt

### Quantitative reverse transcription-polymerase chain reaction (qRT-PCR)

In this study, the gene expressions of the α1 type I collagen gene (Col1α1), the α1 type III collagen gene (Col3α1) , Col5α1 and Col5α2 of injured tendon tissue, normal tendon tissue and tissue engineered tendon were detected. The injured tendon tissue was removed from the Achilles tendon of Sprague-Dawley rats, which had suffered a gap wound model (1 mm in width and 4 mm in length) and was repaired with no treatment after one week [44]. The normal tendon tissue was removed from the Achilles tendon of Sprague-Dawley rats.

In the siRNAs treated experiment, tenocytes were cultured for 48 h after transfection. Real-time PCR was used to detect the mRNAs encoding Col5α1, Col5α2, Col1α1, Col3α1, decorin and GAPDH (n = 3). Total cellular RNA was isolated by lysis in Trizol (Invitrogen, Carlsbad, CA, USA) followed by one-step phenol chloroform-isopropyl alcohol extraction as described in the protocol. For real-time PCR, 2 µg of total RNA from each sample was reversed transcribed using mMLV reverse transcriptase (Invitrogen, Carlsbad, CA, USA). 1/1000 of the reverse transcription reaction was used for subsequent qRT-PCRs, which were set up using TaKaRa Universal PCR Master Mix on an ABI 7900 HT Real-Time PCR System (Applied Biosystems). Target genes were normalized against the housekeeping gene β-actin. Specific primers are shown in Table 2.

**Table 2:** Specific primers used for real-time PCR.

Gene	Sense (5′-3′)	Antisense (5′-3′)
Col5α1	GGACTCGGCGGAACATT	GGAGTTGAGGGAACCAAAGAT
Col5α2	TGTGCGGGGAAGTGTAG	CCAAGAGCAGCAGTAAGAT
Col1α1	TGGATGGCTGCACGAGT	TTGGGATGGAGGGAGTTTA
Col3α1	GCCTCCCAGAACATTACATA	CAATGTCATAGGGTGCGATA
Decorin	ACCACAGTCCATGCCATCAC	AGACTCACGGCAGTGTAGGA
Gapdh	GCAAGTTCAACGGCACAG	CGCCAGTAGACTCCACGAC
β-Actin	AAGATGACGCAGATCATGTTGAG	AGGAGGAGCAATGATCTTGATCTT

### Immunofluorescence staining and quantitative analysis

Immunofluorescence staining was used to detect procollagen type V α1 and α2 chains expression in protein level after treated with specific siRNAs. Tenocytes were grown on the bottom of plastic dishes containing growth medium, and were treated with specific siRNAs. After 48 h, cells were fixed in 4% paraformaldehyde in PBS (pH 7.4) for 15 minutes and then immersed in blocking solution containing 1% bovine serum albumin and 0.3% Triton X-100 for 1 h at room temperature. After washing 3 times in PBS, cells were incubated overnight at 4°C with rabbit and goat polyclonal antibodies against Col5α1 and Col5α2 respectively (1∶100, Santa Cruz, USA), followed by incubation with fluorescence isothiocyanate-conjugated (green,1∶200, Beyotime, China) and cyanine 3-conjugated (red, 1∶30, Sigma, USA) secondary antibody for 1 h. Cells treated with transfection agent only were used as control group. Finally, the incubated cells were rinsed in PBS and observed under a microscope (Olympus, Tokyo, Japan) (n = 3). After immunofluorescence experiment, three pictures of separate view fields were chosen randomly for each sample, and then Image-Pro Plus 5.1.2 was used to quantify the expression level of each procollagen by calculating the related color density.

### MTT assay

Cell survival was determined by MTT assay 1 day and 3 days after treatment of Col5α1 and Col5α2 siRNAs (n = 3). This measure was based on the cleavage of MTT into a blue-colored product (formazan) by the mitochondrial enzyme succinate dehydrogenase [45]–[47]. MTT (50 µg/ml) was added and incubated at 37°C for 4 h. Then, the MTT solution was discarded and 150 µL of dimethyl sulfoxide was added to dissolve the formazan crystals. The plates were then fully shaken to dissolve the formazan crystals formed. The optical density was measured at 570 nm using a Multiskan Spectrum ELIASA (Thermo Labsystems, Waltham, MA, USA).

### Cell morphological assessment and DAPI staining

To detect morphological evidence of apoptosis, cell nuclei were visualized after DNA staining with the fluorescent dye DAPI. Briefly, cells were seeded on coverslips in 24-well tissue culture plates and treated with specific siRNA oligonucleotides. After 3 days, the morphology of cells was monitored under an inverted light microscope. Cells were fixed with 4% paraformaldehyde for 20 min, washed with PBS, and then incubated with DAPI (1 µg/mL) for 10 min. After washing with PBS, cells were observed using fluorescent microscopy (Olympus, Tokyo, Japan) at a peak excitation wavelength of 340 nm (n = 3).

### Fabrication of the tissue engineered tendon

To conveniently detect the collagen fibril synthesis in *in vitro* study, a scaffold-free tissue engineered tendon was developed with tendon cell sheets. Tenocytes were cultured in high glucose DMEM with 10% (v/v) FBS. To prevent cell senescence and promote extracellular matrix synthesis 50 µg/mL of ascorbic acid was added to the medium. Cells proliferated rapidly and formed coherent cellular sheets within 2 weeks. The cell sheets could be detached from the substratum by applying a small roll-up force [24]. The cell sheets were called tissue engineered tendon and used for gene expression andcollagen content detection , as well as transmission electron microscopy analysis in subsequent experiments.

### Histological examination and Masson trichrome staining

To validate whether the tissue engineered tendon mimics tissue *in vivo*, the morphology property of tissue engineered tendon was detected. The engineered tendon without siRNA treatment was fixed in 10% neutral buffered formalin, dehydrated through an alcohol gradient, cleared, and embedded in paraffin. Histological sections (10 µm) were prepared using a microtome and subsequently stained with hematoxylin-eosin and Masson trichrome according to standard procedures to examine the general appearance of the collagen fibrils [48], [49] (n = 3).

### Determination of collagen content of cell sheet

For collagen synthesis studies, cells were transfected with specific siRNA oligonucleotide every four days until a cell sheet was formed. The cells were washed with PBS three times. The amount of deposited collagen in the cell sheet was quantified using a collagen assay kit and following the manufacturer's protocol, which was a colorimetric-based hydroxyproline assay used to estimate total collagen content and concentration(n = 3, Jiancheng Ltd., Nanjing, China) [48]. The content was measured using the Multiskan Spectrum ELIASA at 550 nm.

### Transmission electron microscopic analysis

To verify that extracellular matrix deposited in the tissue engineered tendon, transmission electron microscopy (TEM) was used to assess the diameter of the collagen fibrils and fibril alignment. Briefly, samples were prefixed with 2.5% glutaraldehyde for 2 h at 4°C and washed twice with PBS at 4°C followed by postfixation with 1% osmic acid for 2 h at 4°C. After two washes in PBS, the samples were dehydrated in an ethanol gradient and dried to a critical point. Then the samples were mounted and sputter-coated with gold for viewing in a TEM (Quanta 10 FEI)[50]. About 500 collagen fibrils were measured in each sample to obtain a true representation of the fibril diameter distribution (n = 3).

### Ratio coculture experiments

To find an optimal level of collagen type V in regulating collagen fibrillogenesis, we duplicated the experiment of coculturing Col5α1 siRNA treated tenocytes with normal tenocytes in different ratios. The tenocytes were treated with Col5α1 siRNA three times every 4 days and then detached using 0.25% trypsin and 0.1% ethylenediaminetetraacetic acid (EDTA). Finally, those treated tenocytes were cocultured with untreated tenocytes in a ratio of 1∶0, 1∶0.5, 1∶1, 1∶ 2 or 1∶3. Also, we cultured a control group of normal tenocytes. After two weeks, cell sheets were formed and the TEM analysis was detected (n = 3).

### Statistical analysis

The results are presented as mean ± standard deviation of the 3 samples from each group. The significance of differences was analyzed by Q-test of one-way ANOVA and p <0.05 was considered as statistically significant.
